# Associations between neighborhood walkability and walking following residential relocation: Findings from Alberta's Tomorrow Project

**DOI:** 10.3389/fpubh.2022.1116691

**Published:** 2023-01-16

**Authors:** Gavin R. McCormack, Mohammad Javad Koohsari, Jennifer E. Vena, Koichiro Oka, Tomoki Nakaya, Jonathan Chapman, Ryan Martinson, Graham Matsalla

**Affiliations:** ^1^Cumming School of Medicine, University of Calgary, Calgary, AB, Canada; ^2^Faculty of Kinesiology, University of Calgary, Calgary, AB, Canada; ^3^School of Architecture, Planning and Landscape, University of Calgary, Calgary, AB, Canada; ^4^School of Knowledge Science, Japan Advanced Institute of Science and Technology, Nomi, Japan; ^5^Faculty of Sport Sciences, Waseda University, Tokorozawa, Japan; ^6^Cancer Care Alberta, Alberta Health Services, Calgary, AB, Canada; ^7^Graduate School of Environmental Studies, Tohoku University, Sendai, Japan; ^8^Public Space and Mobility Policy, Planning and Development Services Department, Calgary, AB, Canada; ^9^Toole Design Group, Calgary, AB, Canada; ^10^Mental Health Promotion and Illness Prevention, Alberta Health Services, Calgary, AB, Canada

**Keywords:** urban design, longitudinal, physical activity, built environment, urban form

## Abstract

**Introduction:**

Cross-sectional studies consistently find that the neighborhood built environment (e.g., walkability) is associated with walking. However, findings from the few existing longitudinal residential relocation studies that have estimated associations between changes in neighborhood built characteristics and walking are equivocal. The study objective was to estimate whether changes in neighborhood walkability resulting from residential relocation were associated with leisure, transportation, and total walking levels among adults.

**Methods:**

This study included longitudinal data from the “Alberta's Tomorrow Project”—a province-wide cohort study (Alberta, Canada). The analysis included data collected at two time points (i.e., baseline and follow-up) from 5,977 urban adults. The International Physical Activity Questionnaire (IPAQ) captured self-reported walking. We estimated neighborhood walkability, an index capturing intersection, destination, and population counts for the 400 m Euclidean buffer around participants' homes. Using household postal codes reported at baseline and follow-up, we categorized participants into three groups reflecting residential relocation (“non-movers:” *n* = 5,679; “movers to less walkability:” *n* = 164, and; “movers to more walkability:” *n* = 134). We used Inverse-Probability-Weighted Regression Adjustment to estimate differences [i.e., average treatment effects in the treated (ATET)] in weekly minutes of leisure, transportation, and total walking at follow-up between residential relocation groups, adjusting for baseline walking, sociodemographic characteristics, and walkability. The median time between baseline and follow-up was 2-years.

**Results:**

The three residential relocation groups mainly included women (61.6–67.2%) and had a mean age of between 52.2 and 55.7 years. Compared to “non-movers” (reference group), weekly minutes of transportation walking at follow-up was significantly lower among adults who moved to less walkable neighborhoods (ATET: −41.34, 95 CI: −68.30, −14.39; *p* < 0.01). We found no other statistically significant differences in walking between the groups.

**Discussion:**

Our findings suggest that relocating to less walkable neighborhoods could have detrimental effects on transportation walking to the extent of adversely affecting health. Public health strategies that counteract the negative impacts of low walkable neighborhoods and leverage the supportiveness of high walkable neighborhoods might promote more walking.

## Introduction

Encouraging adults to participate in regular physical activity is important as it can protect against numerous modifiable chronic health conditions (e.g., cardiovascular disease, type II diabetes, hypertension, metabolic syndrome, overweight and obesity, cancer, and depression) (1, 2) and premature mortality (3, 4). There is a dose-response relationship between physical activity and health, meaning that even small amounts of moderate-intensity physical activity daily (e.g., walking) can provide health benefits (5). From a public health perspective, walking is a prime intervention target for encouraging the accumulation of physical activity because walking is a safe and innate behavior that most adults can efficiently perform as part of their everyday activities within various physical settings (6). Furthermore, walking is one of the most commonly undertaken physical activities (7, 8) and an essential contributor to the accumulation of total physical activity (9, 10). However, in Canada, less than one-third of adults walk regularly (≥4 times per week) (10), and on average, they accumulate < 5,000 steps per day (11). There is an urgent need to identify effective population-level interventions that encourage more walking among Canadian adults.

Neighborhoods are popular settings where adults walk (12–15). The creation of walkable neighborhood built environments is an intervention strategy that can increase walking and increase physical activity (16–18), social connectedness (19), and improve health (20, 21). Walkable neighborhoods include several built attributes such as connected street layouts, pedestrian infrastructure, amenities, and safety, making walking an easy and convenient option (22). Cross-sectional evidence demonstrates that neighborhoods with higher walkability are associated with more walking (23, 24). However, cross-sectional studies provide no evidence with regard to temporality, limiting their ability to assess causality. It remains unclear whether associations between neighborhood walkability and walking result from neighborhood self-selection (seeking out walkable neighborhoods to fulfill walking preferences) or if exposure to a walkable neighborhood changes walking behavior. Recognizing this limitation, longitudinal studies (retrospective and prospective residential relocation studies and natural experiments) estimating the associations between changes in built environment exposure and physical activity have recently emerged (17, 18, 25, 26). Residential relocation studies, in particular, offer an opportunity to estimate the temporal relations between the built environment and physical activity and can account for residual confounding because participants serve as their controls (26). Moreover, residential relocation interrupts normal or habitual behavioral patterns, of which some of this behavior change might be due to exposure to a different built environment (26, 27).

Findings from residential relocation studies provide only modest and often mixed evidence for an association between the built environment and physical activity (26). Most residential relocation studies to date have been undertaken in the US, Australia, and in European countries, with few studies conducted in Canada (26). The geographical differences in physical activity and the built environment strengthens the need for more Canadian specific studies investigating associations between neighborhood walkability and walking (18). Moreover, prospective residential relocation studies undertaken to date have mostly focussed on associations and found consistent results in relation to the associations between land uses, destinations, and transportation and walking (26, 28) with fewer studies examining changes in exposure to overall neighborhood walkability and walking (26). In a residential relocation study in Canada (average follow-up period of 10 months), Adhikari et al. (29) found that an increase in walkability (residential density, commercial floor area ratio, land use mix, and intersection density combined) was associated with an increase in non-work transport-related walking trips adjusting for changes in neighborhood and travel preferences and life events. In another prospective residential relocation study (12 year follow-up) undertaken in Canada, Wasfi et al. (30) found that relocation to a more walkable neighborhood (measured using Walk Score^®^) was associated with an increase in the likelihood of participating in transportation walking. In a residential relocation study in the UK (2 year follow-up), Clary et al. (31) found that one standard deviation increase in walkability (i.e., connectivity, land use mix, and residential density combined) was associated with an increase of ~300 steps and 1.7 min of MVPA per day. In contrast, in a prospective residential relocation study in the US (6 year follow-up), Braun et al. (32) found no significant associations between changes in walkability (population density, connectivity, and food and physical activity resources combined) and participation and frequency of overall walking among movers. A residential relocation study (“RESIDE”) undertaken in Western Australia, which spanned 10-years found adults exposed to neighborhoods that incorporated liveable urban design features (i.e., safe, convenient pedestrian-friendly, access to shops, transit, and parkland) in general undertook more local walking (in particular transportation walking), and had a stronger sense of community and improved mental health (28).

Residential relocation studies to date have generated mixed findings regarding associations between the built environment and physical activity, few have investigated associations specifically between neighborhood walkability and walking, and the extent to which walkability and walking for different purposes are temporally related remains unclear (26). Further, there is a need for context specific evidence (i.e., Canadian data) to better inform local urban planning and public policy and practice decision-making. Therefore, the aim of our study was to investigate whether changes in neighborhood walkability resulting from residential relocation were associated with leisure, transportation, and total walking among adults in the Canadian context.

## Methods

### Study and sample design

Previous articles have described the methodological details of the Alberta's Tomorrow Project (ATP) (33, 34). Briefly, ATP is a longitudinal, province-wide study that began in Alberta (Canada) in 2000. From 2000 to 2008, a random sample of adults aged 35–69 years (*n* = 63,486) were invited to complete a health and lifestyle questionnaire (HLQ), of which *n* = 31,072 responded. In 2008, *n* = 20,707 participants completed a first follow-up survey and between 2009 and 2015, *n* = 15,963 completed a second follow-up survey (34). We undertook secondary (longitudinal) analysis of ATP data that were collected in 2008 and from 2009 to 2015 (herein referred to as “baseline” and “follow-up,” respectively) because the walking outcome measures of interest were consistent between these two surveys. Participant's residential addresses were collected at each survey however, to comply with ethics and to maintain participant anonymity only postal codes could be used for analyses (e.g., linking with built environment data). In our analysis, we included participants from urban areas only, identified from the Forward Sortation Area (FSA) information contained in the first 3-digits of their 6-digit residential postal codes. Rural postal codes include a zero in the second position of the FSA and denotes an area where there are no letter carriers (mail delivered to a post office or postal box). We included only participants from urban areas only because there are urban-rural differences in land use and transportation planning processes, built characteristics may exert different effects on walking in urban vs. rural neighborhoods (35), and in Canadian there are urban-rural differences in the prevalence physical inactivity (36).

Further, we included data from participants that provided complete data on the variables of interest for both the baseline and follow-up surveys, with which built environment data were available and linkable to their survey data (*n* = 5,977). The median follow-up time between the baseline and follow-up surveys was 2 years. The University of Calgary Conjoint Health Research Ethics Board approved the acquisition and analysis of ATP data for this study (REB17-1466).

### Variables

#### Self-reported physical activity

The International Physical Activity Questionnaire (IPAQ) (37), captured leisure and transportation walking at baseline and follow-up. Leisure walking included walking undertaken for recreation, sport or leisure and transportation walking included walking undertaken to go from place to place. Participants reported the number of days in the past week they undertook at least 10-min of leisure or transportation walking. Participants also reported time spent undertaking walking during a typical day. We multiplied reported days by reported daily minutes to estimate weekly minutes of leisure, transportation, and total walking (i.e., leisure plus transportation walking). As recommended (38), we truncated each walking outcome to 180-min per day to remove outliers and adjust for over-reporting.

#### Neighborhood walkability

The walkability index used in our analysis has been described elsewhere (39). We geocoded all Alberta 6-digit urban postal codes for 2008–2015 (*n* = 77,602–84,115) to create points. Canadian urban postal codes provide a reasonable approximation of household location, when complete street address information is not available (e.g., 50% of postal codes are located within a 69 m, and 88% of postal codes are located within 200 m, of the true household address location) (40). Using Geographical Information Systems, we created 400 m Euclidean buffers (polygons) around each geocoded postal code point. Neighborhoods were defined by a 400 m buffer boundary that captured neighborhood built characteristics located within about a 5–10 min walking distance from home (41). Overlaid with a street network file (CanMap Streetfiles and Route Logistics, DMTI Spatial Inc.), we calculated the count of 3-way and 4-way intersections within each buffer. Using the Enhanced Points of Interest (DMTI Spatial Inc.) and available Standard Industry Codes, we calculated the count of business destinations (e.g., hardware stores, department stores, grocery stores, restaurants, banks, libraries, laundry stores, stationery stores, liquor stores, jewelry stores, barbershops, museums, schools, colleges, and universities) within each buffer. To calculate population counts, we overlaid dissemination area census data from Statistics Canada (years 2006, 2011, and 2016) onto the buffers. Dissemination areas (DA) are the smallest standard geographical unit available for research purposes from Statistics Canada (each DA includes ~400–700 persons) (42). DAs have irregular-shaped boundaries that often follow roads or other features (e.g., railways and water features); therefore, they did not match the geographical shape of the circular neighborhood buffers. Thus, the total population count assigned to each buffer was based on a weighted sum that reflected the proportion of geometric overlap between each DA boundary and the buffer (i.e., multiple DAs could overlap a single buffer). For non-census years, we imputed population counts using the averages for years in which census data were available.

For each year (2008–2015) across all buffers, we converted raw counts for intersections (3-way and 4-way), destinations, and population to *z*-scores. The *z*-scores were summed to derive a walkability score (WS) for each buffer [WS = [0.5 × *z* (3-way intersections)] + *z* (4-way intersections) + *z* (destinations) + *z* (population)]. Higher positive scores reflected more walkability, while higher negative scores reflected less walkability. Relative to 4-way intersections, 3-way intersections contribute less to street connectivity and may offer less support for walking (43–45). Therefore, we down-weighted the contribution of 3-way intersections to the WS. The walkability score (estimated for 77,597 postal codes using 2,008 values) was positively correlated with each of the individual built environment variables (i.e., 3-way intersections *r* = 0.400; 4-way intersections *r* = 0.753; destinations *r* = 0.794, and; population count *r* = 0.605). We also found the WS to have concurrent validity compared against Walk Score^®^, a valid, widely-used, measure of walkability (46, 47) (*r* = 0.648; estimate for 81,114 postal codes using 2012 values). The intra-class correlation (ICC) for WSs estimated for postal codes available for all years from 2008 to 2015 (ICC = 0.974) showed that the estimated WS were relatively stable during this period.

We temporally matched participant survey data (baseline and follow-up) with buffer WSs using 6-digit postal codes and created three residential relocation groups. Among movers, the direction of the absolute difference between baseline (origin neighborhood) and follow-up (destination neighborhood) walkability scores, regardless of magnitude, was used to categorize participants as having relocated to either a: (1) less walkable neighborhood or; (2) more walkable neighborhood. Non-movers constituted the third category, regardless of the direction of change in walkability estimated between the baseline and follow-up surveys. Including a non-mover control group is vital for accounting for changes in walking that might have occurred had those moving not relocated neighborhood (i.e., the counterfactual) and for isolating the effect of a change in walkability (e.g., increase or decrease in walkability) on walking.

#### Sociodemographic characteristics

Sociodemographic variables from the baseline survey included sex, age, children under 18 years of age at home, highest educational attainment, annual gross household income, marital status, employment status, and dwelling type in origin neighborhood (i.e., single dwelling, duplex or row housing, apartment, and other). The season of survey completion was captured.

### Statistical analysis

We used descriptive statistics (mean, standard deviation, and frequencies) and inferential statistics (One-way Analysis of Variance—ANOVA, and Pearson's chi-square) to estimate the differences in baseline sociodemographic and walking variables between the three residential relocation groups (i.e., non-movers, moved to less walkability, and moved to more walkability). ANOVA (with Tukey-Kramer *post hoc* tests) estimated between-group differences in the walkability of the origin and destination neighborhoods (or change over time in the origin neighborhood walkability only for non-movers) for the three residential relocation groups. Dependent (paired) *t*-tests estimated within-group differences between the walkability of origin and destination neighborhoods for each residential relocation group. Using pooled data (ignoring residential relocation groupings), we performed generalized linear regression (Gaussian distribution with identity link) to estimate the slope coefficients (b) for the baseline cross-sectional associations between walking outcomes and WS, adjusting for covariates (i.e., sex, age, number of children under 18 years of age at home, highest educational attainment, annual gross household income, marital status, employment status, and dwelling type).

We undertook analysis to estimate the average treatment effect on the treated (ATET)—i.e., the estimated difference in leisure, transportation, and total walking between those who moved to less walkability and those who moved to more walkability compared with non-movers. For analytical purposes, we regarded non-movers as a “control” group and those relocating to less or more walkability as the “treatment” groups. Estimating these differences relative to non-movers (control group) accounted for the change in walking behavior among the treatment groups that resulted from factors other than residential relocation and change in walkability exposure.

We used treatment effects models to estimate the ATET. We used the *teffects and ipwra* Stata commands (48) to derive inverse-probability-weighted regression adjusted estimates to optimize the balance in baseline covariates between the three residential relocation groups and to compute the average treatment-level predicted outcomes (leisure, transportation, and total walking). This approach was taken to make the three residential relocation groups conditionally exchangeable (49), allowing for the average causal effect of relocating to either a less or more walkable neighborhood to be estimated from the contrasts in the average predicted outcomes between these two (treatment) groups relative to non-movers (control group). To compute the inverse-probability-weights, a treatment model (multinomial logit) was first estimated in which the residential relocation group was regressed onto the baseline covariates (i.e., sex, age, number of children under 18 years of age at home, highest educational attainment, annual gross household income, marital status, employment status, dwelling type, WS in origin neighborhood, and total walking minutes). We applied the inverse-probability weights to regression (outcome) models and estimated treatment-specific predicted follow-up walking outcomes for each participant. To control for the influence of seasonality on physical activity (50) and, in particular, walking within the Canadian context (51), we included the season during which the participant returned the follow-up survey as a covariate in the regression models. The mean walking outcomes were calculated for each residential relocation group (treatment-specific predicted outcomes), and contrasts were performed to calculate the differences in the means between the two treatment groups relative to non-movers (control group) to provide the treatment-specific ATET estimates.

STATA's *tebalance summarize* and *teffects overlap* commands facilitated covariate balance checking between the three residential relocation groups. We assessed the balance of each baseline covariate using the average standardized absolute mean difference (SMD) and variance ratio (VR) and assessed the visual overlap in the group covariate distributions using box plots and cumulative distribution functions (52). Covariates of perfectly balanced groups have an SMD = 0 and VR = 1; however, we considered groups to be sufficiently balanced if the SMD was | < 0.1| and the VR was 0.5–2 for all covariates (53, 54). We estimated bootstrapped standard errors (1,000 repetitions with replacement) and 95% confidence intervals (95 CI) for the generalized linear and treatment effect models. We considered *p*-values < 0.05 as statistically significant. We undertook the analysis using Stata/SE 15.1 (StataCorp LLC, College Station, Texas, USA).

## Results

### Sample characteristics

The sample (*n* = 5,977) consisted mostly of participants that were female, married, employed and residing in single-dwelling homes and had completed post-secondary education (Table 1). Approximately 5% (*n* = 298) of our sample relocated neighborhood between the baseline and follow-up surveys. Non-movers, movers to less walkability, and movers to more walkability significantly differed (*p* < 0.05) concerning their baseline age, marital status, employment status, and dwelling type. Mean weekly minutes of leisure, transportation, and total walking at baseline and follow-up did not significantly differ by residential relocation group, except for follow-up transportation walking (i.e., movers = 119.5, movers to less walkability = 83.5, and movers to more walkability = 128.6; *p* < 0.05; Table 1). Among all participants from baseline to follow-up (pooled), we found, on average, significant decreases in weekly minutes of leisure walking (Δ = −9.28, 95 CI: −4.26, −14.29, *p* < 0.001) and total walking (Δ = −8.77, 95 CI: −0.94, −16.59, *p* = 0.028) but not transportation walking (Δ = 0.83, 95 CI: −4.56, 6.22, *p* = 0.764).

**Table 1:** Sample characteristics by residential relocation group.

**Baseline sociodemographic characteristics**	**Residential relocation group**		
	**Non-mover**	**Moved to a less walkable neighborhood**	**Moved to a more walkable neighborhood**
	**(*n* = 5,679)**	**(*n* = 164)**	**(*n* = 134)**
	**Estimate**	**Estimate**	**Estimate**
**Sex (female %)**	61.6	64.6	67.2
**Age [mean, (SD)]** ^*^	55.7 (9.1)	51.6 (8.2)	52.2 (9.6)
**Number of children [mean (SD)]** **Education attained (%)**	0.5 (0.9)	0.5 (1.0)	0.5 (0.9)
High school or less	17.9	17.7	19.4
Some post-secondary	23.6	20.7	26.1
Completed post-secondary	55.5	61.6	54.5
**Annual household income (%)**			
≤ $49,999	17.2	25.6	21.6
$50,000–99,999	30.6	28.7	29.1
$100,000–149,999	23.8	19.5	22.4
$150,000–199,999	10.6	7.9	10.4
≥$200,000	10.2	14.0	11.2
Don't know/refused	7.6	4.3	5.2
**Marital status (married/defacto %)** ^*^	73.2	62.2	64.9
**Dwelling type in origin neighborhood (%)** ^*^			
Single dwelling home	79.3	62.8	65.7
Duplex or row housing	10.9	15.8	10.4
Apartment	7.6	16.5	19.7
Other	2.2	4.9	5.2
**Employment status (employed %)** ^*^	67.5	78.0	77.6
**Walking minutes per week [mean (SD)]**			
Baseline transportation walking	118.0 (180.0)	100.7 (169.5)	134.0 (197.5)
Follow-up transportation walking^*^	119.5 (188.4)	83.5 (133.5)	128.6 (219.4)
Baseline leisure walking	123.8 (179.0)	109.1 (173.9)	97.3 (135.7)
Follow-up leisure walking	113.8 (167.7)	103.6 (167.5)	112.3 (175.1)
Baseline total walking	243.5 (285.6)	214.9 (285.5)	231.3 (268.0)
Follow-up total walking	234.8 (286.4)	187.0 (235.0)	240.8 (317.4)

SD, standard deviation.

Between group (relocation status) differences in continuous variables compared using ANOVA and in categorical variables compared using Pearson's chi-square.

^*^Statistically significant (*p* < 0.05).

Among the entire sample, mean (SD; median; minimum; maximum) walkability was −0.09 (2.15; −0.31; −4.32; 11.84) at baseline and −0.07 (2.19; −0.29; −4.37; 12.04) at follow-up. Mean walkability at baseline was significantly different between the residential relocation groups (Table 2). Notably, participants who moved to neighborhoods with less walkability came from origin neighborhoods that were significantly (*p* < 0.01) more walkable (WS = 0.86) compared with those who moved to more walkability (WS = −0.95) or non-movers (WS = −0.10). Among non-movers, change in walkability between the baseline and follow-up significantly improved (Δ = 0.03; 95 CI: 0.02, 0.05, *p* < 0.001), albeit by a smaller magnitude relative to the absolute changes in walkability observed among those who moved to less (Δ = −2.14; 95 CI: −2.43, −1.85) and more walkable neighborhoods (Δ = 2.40; 95 CI: 2.01, 2.78; Table 2).

**Table 2:** Walkability scores for origin and destination neighborhoods by residential relocation group.

	**Residential relocation group**		
	**Non-mover**	**Moved to a less walkable neighborhood**	**Moved to a more walkable neighborhood**
	**(*****n*** = **5,678)**	**(*****n*** = **164)**	**(*****n*** = **134)**
**Origin neighborhood walkability (baseline)**			
Mean (95 CI)	−0.10 (-0.16, −0.05)^a, b^	0.86 (0.53, 1.19)^a, c^	−0.95 (−1.32, −0.59)^b, c^
Standard deviation	2.14	2.31	2.13
Minimum and maximum	−4.32, 11.84	−3.11, 8.08	−4.22, 8.26
Median (25 and 75th percentiles)	−0.32 (−1.44, 1.07)	0.61 (−0.98, 2.20)	−1.13 (−2.31, 0.06)
**Destination neighborhood walkability (follow–up)**			
Mean (95 CI)	−0.07 (−0.13, −0.01)^a, b^	−1.28 (−1.61, −0.95)^a, c^	1.44 (1.08, 1.81)^b, c^
Standard deviation	2.16	1.72	2.95
Minimum and maximum	−4.37, 12.04	−4.33, 4.15	−3.57, 11.64
Median (25 and 75th percentiles)	−0.28 (−1.39, 1.11)	−1.36 (−2.52, −0.36)	1.07 (−0.27, 2.14)
**Absolute change in walkability (follow–up minus baseline)**			
Mean (95 CI)	0.03 (0.02, 0.05)^*^	−2.14 (−2.43, −1.85)^*^	2.40 (2.01, 2.78)^*^
Standard deviation	0.61	1.89	2.24
Minimum and maximum	−3.71, 4.29	−9.59, −0.01	0.01, 10.98
Median (25 and 75th percentiles)	−0.04 (−0.23, 0.22)	−1.57 (−2.99, −0.77)	1.77 (0.80, 3.16)

Same superscript (^a, b, c^) represents statistically significant (p < 0.01) pairwise between group differences in walkability (ANOVA and Tukey-Kramer *post-hoc* tests).

^*^Statistically significant (p < 0.001) within group differences in walkability (paired t-tests).

### Baseline cross-sectional associations between walkability and walking outcomes

Adjusting for all covariates, WS was positively associated with baseline weekly minutes of transportation walking (*b* = 3.17; 95 CI: 0.82, 5.53; *p* = 0.008), but not with baseline minutes of leisure walking (*b* = −1.33; 95 CI: −3.59, 0.94; *p* = 0.251) or total walking (*b* = 1.89; 95 CI: −1.60, 5.38; *p* = 0.288).

### Covariate balance between the residential relocation groups

**Figure 1:**
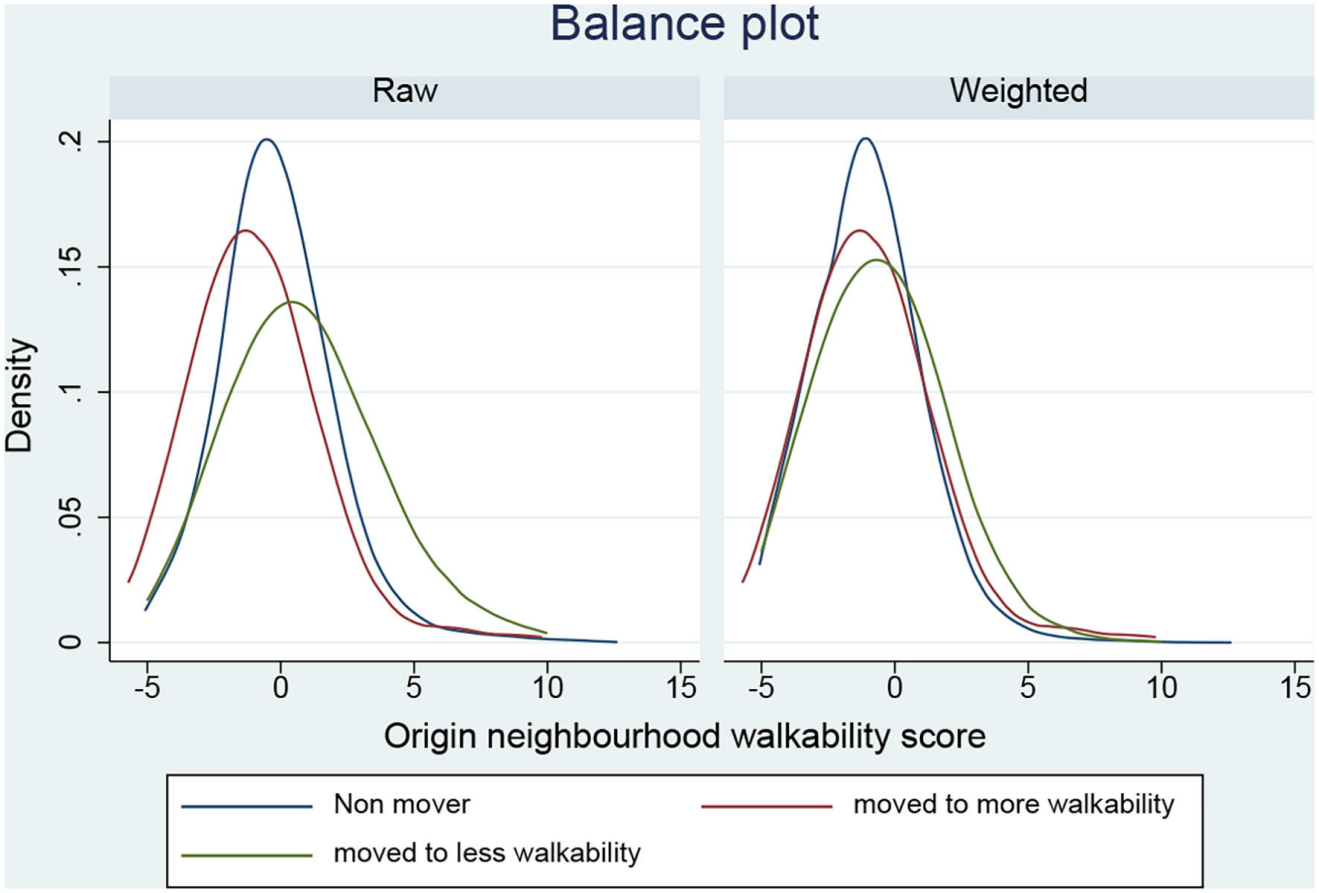
Balance in origin neighborhood walkability scores between residential relocation status groups before and after weighting.

The inverse-probability-weights generated from the treatment model improved covariate balance between the three residential relocation groups concerning the SMDs (unweighted: range = −0.473 to 0.433 vs. weighted: range = −0.117 to 0.247) and VRs (unweighted: range = 0.598 to 2.737 vs. weighted: range = 0.738–1.298; Table 3; Figure 1). Although improved after weighting, the SMD for baseline walkability (SMD from 0.433 to 0.247) did not meet the criteria for establishing covariate balance (i.e., SMD = | < 0.1| and the VR = 0.5–2; Table 3). As a result, we doubly-adjusted for baseline walkability by including it as covariate in the treatment effect model.

**Table 3:** Covariate balance of baseline covariates before and after weighting between residential relocation groups.

	**Standardized differences**		**Variance ratio**	
	**Unweighted**	**Weighted**	**Unweighted**	**Weighted**
**Moved to more walkable neighborhood**				
Total walking	−0.044	−0.006	0.880	0.945
Sex	−0.116	0.001	0.939	1.000
Age	−0.369	−0.001	1.105	1.292
Income (cat2)	−0.033	0.007	0.978	1.006
Income (cat3)	−0.034	0.005	0.964	1.007
Income (cat4)	−0.005	0.002	0.995	1.005
Income (cat5)	0.032	−0.001	1.094	0.997
Income (cat6)	−0.095	0.005	0.714	1.019
Education (cat2)	0.059	−0.003	1.079	0.996
Education3 (cat3)	−0.081	0.018	1.029	0.997
No. children	0.037	0.005	1.072	1.054
Marital status	−0.179	0.010	1.169	0.994
Employment	0.228	0.010	0.798	0.986
Walkability	−0.399	0.014	0.995	1.152
Dwelling (cat2)	−0.014	0.009	0.972	1.023
Dwelling (cat3)	0.329	−0.008	2.161	0.988
Dwelling (cat4)	0.163	−0.009	2.373	0.966
**Moved to less walkable neighborhood**				
Total walking	−0.100	−0.078	1.000	1.251
Sex	−0.063	−0.029	0.972	0.978
Age	−0.473	−0.010	0.820	0.950
Income (cat2)	−0.043	0.112	0.968	1.094
Income (cat3)	−0.105	−0.117	0.870	0.837
Income (cat4)	−0.092	0.037	0.775	1.097
Income (cat5)	0.117	0.035	1.325	1.086
Income (cat6)	−0.140	−0.011	0.589	0.956
Education (cat2)	−0.068	0.038	0.918	1.040
Education3 (cat3)	0.063	0.005	0.980	0.999
No. children	0.089	−0.039	1.239	1.255
Marital status	−0.236	0.012	1.205	0.993
Employment	0.238	0.024	0.786	0.968
Walkability	0.433	0.247	1.173	0.738
Dwelling (cat2)	0.146	0.111	1.384	1.298
Dwelling (cat3)	0.272	0.084	1.956	1.130
Dwelling (cat4)	0.148	−0.044	2.220	0.834

Number of unweighted observations: Non-movers (n = 5,679); Moved to more walkable neighborhood (*n* = 134); Moved to less walkable neighborhood (*n* = 164).

Number of observations after applying inverse probability weighting: Non-movers (*n* = 2,054.4); Moved to more walkable neighborhood (n = 2,042.4); Moved to less walkable neighborhood (*n* = 1,879.8).

### Differences in walking behavior by residential relocation group (ATET)

Compared to non-movers, weekly minutes of transportation walking was significantly lower at follow-up among those who moved to a less walkable neighborhood (−43.48 min/week; 95 CI: −68.17, 17.78, *p* < 0.01; Table 4). Furthermore, the difference in weekly minutes of total walking at follow-up between non-movers and those who moved to a less walkable neighborhood approached statistical significance (−50.86; 95 CI: −102.67, 0.99, *p* = 0.054). We found no other statistically significant between-group differences in walking.

**Table 4:** Differences (ATET) in weekly minutes per week of walking duration post relocation by relocation group.

	**Group**	**Transportation walking**		**Leisure walking**		**Total walking**	
		**b (95 CI)**	***P***	**b (95 CI)**	***P***	**b (95 CI)**	***p***
**Naïve estimate** ^a^							
Non-mover	Control	0		0		0	
Moved to more walkable neighborhood	Treatment 1	9.07 (−29.06, 47.21)	0.641	−1.61 (−30.92, 27.70)	0.914	6.01 (−47.84, 59.87)	0.827
Moved to less walkable neighborhood	Treatment 2	−36.03 (−56.54, −15.53)	0.001	−10.28 (−36.35, 15.78)	0.439	−47.77 (−83.19, −12.34)	0.008
**Adjusted estimates** ^b^							
Non-mover	Control	0		0		0	
Moved to more walkable neighborhood	Treatment 1	13.75 (−22.40, 49.89)	0.456	1.43 (−29.84, 32.71)	0.928	14.15 (−39.55, 67.85)	0.604
Moved to less walkable neighborhood	Treatment 2	−43.48 (−69.17, −17.78)	0.001	−6.36 (−49.70, 36.98)	0.774	−50.86 (−102.67, 0.99)	0.054

ATET, Average Treatment Effects in the Treated.

^a^No adjustment for covariates in the treatment or outcome models.

^b^Based on inverse probability weighting to balance covariates between residential relocation groups (treatment model) and inclusion of season and baseline walkability as covariates in the outcome model.

95 CI estimated from bootstrapped standard errors (1,000 replications with replacement).

## Discussion

Residential relocation studies provide a unique opportunity for generating rigorous causal evidence that can inform urban design and public health policy (55, 56). Our study investigated whether a change in neighborhood walkability resulting from residential relocation was associated with leisure, transportation, and total walking levels among adults in the Canadian context. Our findings suggest that relocating to a less walkable neighborhood may have detrimental effects on transport-specific and total walking. Specifically, we found that compared with non-movers, adults moving to less walkable neighborhoods undertook ~41 min less of transportation walking per week. A difference in walking of this magnitude is of clinical relevance, and could have negative physical and mental health consequences (57–60). Our findings are congruent with other studies demonstrating that a change in walkability following residential relocation was associated with transportation walking (29, 30) however; our findings are novel in that we only found a significant difference in transportation walking for those relocating to neighborhoods of less, but not to more walkability. Our cross-sectional and longitudinal findings support the consistent evidence demonstrating that the neighborhood built environment may be more strongly associated with transportation versus leisure walking (18, 23, 24).

A notable finding from this study was that relocating to a less walkable neighborhood may have a greater impact on transportation walking than relocating to a higher walkable neighborhood. The reduction in transportation walking (41 min per week, on average), was in response to an average reduction in walkability (between origin and destination neighborhood) by ~2 standard deviations, independent of the walkability of the origin neighborhood. However, given that walkability was measured as a score which combined three built environment variables (*z*-scores representing transformed counts of intersections, destinations, and population) translating this score into a description of the change in built environment is difficult. Nevertheless, this finding aligns with the general scientific consensus that higher neighborhood walkability is better for walking, and in particular transportation walking. Our novel finding may reflect the mismatch between walking preferences and built environment characteristics that provide opportunities to walk (61, 62). Specifically, adults preferring to walk for transportation but who relocate to a neighborhood that has an unsupportive built environment (i.e., low walkability) may have no option but to walk less (i.e., a discouraging effect). Alternatively, an adult who relocates to a neighborhood with a built environment that is more supportive (i.e., high walkability) have the option to choose to walk for transportation or not walk according to their preferences. Our finding might also suggest that the transportation walking patterns or habits formed while residing in less walkable neighborhoods remain even after relocating to a more walkable neighborhood. That is, those who did not walk for transport in their origin neighborhoods may be less likely to initiate transportation walking after relocating to a more walkable neighborhood.

Unlike transportation walking, we found that walkability was not associated with leisure walking in either the cross-sectional or longitudinal analysis. Notably, weekly minutes of leisure walking was not impacted by relocating neighborhood (whether it be less or more walkable), which might suggest that preferences for and opportunities to undertake this type of walking is less constrained by the neighborhood built environment. Other studies however, have found changes in leisure walking following neighborhood relocation. For instance, improvements in participation of transportation and leisure walking have been reported among adults who relocated to a mixed-use (e.g., more walkable) neighborhood (63). Nevertheless, leisure walking may be more strongly determined by proximal intrapersonal factors (e.g., self-efficacy, intentions, enjoyment, and perceived barriers) with a smaller influence from the built environment (64, 65). Our lack of significant findings regarding leisure walking may also reflect the built environment characteristics included in our walkability measure. The walkability measure did not include built attributes such as parks and pathways that may be important for supporting leisure walking (66). More research on how changes in exposure to individual built characteristics influences walking undertaken for different purposes is needed (26).

Our findings have important implications. For adults considering relocating neighborhood and wanting to continue to walk for transportation, our findings suggest that these adults should seek out new neighborhoods with at least similar or higher levels of walkability relative to their origin neighborhoods. Publically available tools, such as Walk Score^®^, Bike Score^®^, and Transit Score^®^ (available on real estate sites such as Redfin: https://www.redfin.com/) may be useful for identifying new neighborhoods that can support preferences for transport-related physical activity. Moreover, real estate professionals may play an important role in matching home-seekers with neighborhoods that include built characteristics that support their walking preferences (67–69). More importantly, our findings highlight the need for urban development and planning authorities, municipalities, and land developers to increase the supply of newly developed walkable neighborhoods and invest in the redevelopment of existing neighborhoods to increase their supportiveness for transportation walking. Broadening the availability of walkable neighborhoods may reduce the number of people who decrease their transportation walking following relocation as poor design might overshadow people's intentions to walk. Improving the built environment and implementing strategies for improving awareness and education on the importance of walkability among home-seekers is important. Further, our findings lend support for public health strategies (e.g., mass media campaigns and individualized trip planning) that counteract the negative impact of poor urban design in low walkable neighborhoods and capitalize on the supportive pedestrian infrastructure available in walkable neighborhoods.

Our study has several strengths. Our objective-measure of walkability, estimated change in walkability exposure resulting from neighborhood relocation, inclusion of measures of walking for different purposes, statistical adjustment for baseline walking and walkability, inclusion of a control group (“non-movers”), and our two-staged modeling strategy that balanced the observed covariates between the groups prior to treatment effect estimation strengthened internal validity. Non-movers were observed to have a relatively small change in walkability between the baseline and follow-up suggesting that the neighborhood built environment remained relatively stable during the short term (70, 71). Moreover, this temporal stability was also supported by our estimated intra-class correlation for the neighborhood walkability score for years 2008–2015 (ICC = 0.974). Participants included in our study were from urban areas that spanned an entire Canadian province, thus contributing to the representativeness of the sample and our findings.

Our study also has several limitations. The median follow-up time of 2 years, limits our ability to infer whether long-term changes in walking might occur as individuals become more aware, accustomed, and exposed to their new neighborhood surroundings. The walkability variable represented the combination of three built environment variables (intersections, destinations, and population), and while these are important characteristics for supporting walking (16–18), they do not represent all neighborhood built characteristics that might facilitate walking. Examining changes in walking resulting from changes in exposure to neighborhood built characteristics such as transit availability and accessibility, green space, pathways, traffic and personal safety, and aesthetics may be important to consider in future research. Moreover, the small sample of movers did not allow us to examine and test different cut-off scores for change in walkability exposure (i.e., sensitivity analysis), thus we are not able to determine the extent to which magnitude of walkability change affected walking or whether a minimum exposure threshold to elicit a change in walking existed. Walking outcomes were self-reported and may not accurately reflect actual walking (72). Moreover, despite most walking being undertaken close to home (12–15), our walking outcomes were not context-specific (73), thus some of the walking reported likely occurred outside the neighborhood (i.e., at locations further than 400 meters from home). We defined neighborhoods using a 400 m Euclidean buffer representing a 5–10 min walking distance from home (41). Relative to network buffers, Euclidean defined boundaries can result in underestimates of associations between the built environment and walking irrespective of boundary size (74, 75). Speculatively, our walking measure and neighborhood boundary definition likely resulted in conservative estimates of the relationships between neighborhood walkability and walking. The sample included middle-aged to older adults and thus the patterns found in relation to walkability and walking may not generalize to younger adults, limiting the study's generalizability.

Our findings demonstrate that adults who relocate to new neighborhoods that are less walkable than their origin neighborhoods undertake less transportation walking. More longitudinal studies need to investigate the short and long-term changes in walking associated with changes in neighborhood walkability exposure following residential relocation.

## Data availability statement

Data that support the findings of this study are available from the Alberta's Tomorrow Project (https://myatp.ca/) following data requisition approval. Requests to access these datasets should be directed to Alberta's Tomorrow Project (https://myatp.ca/).

## Ethics statement

The studies involving human participants were reviewed and approved by the University of Calgary Conjoint Health Research Ethics Board (REB17-1466). The patients/participants provided their written informed consent to participate in this study.

## Author contributions

GRM, JV, GM, JC, and RM were involved in the conception of the study. JV was involved in the study design and data collection for ATP. GRM, MK, KO, and TN advised on the analysis and interpreted the results. GRM drafted the original manuscript. All authors reviewed and approved the final manuscript.
